# Multimodality Imaging in a Patient with Erdheim Chester Disease with Cardiovascular Involvement

**DOI:** 10.15388/Amed.2025.32.2.18

**Published:** 2025-12-30

**Authors:** Priya Jagia, Vineeta Ojha, Gautam Sharma

**Affiliations:** 1 All India Institute of Medical Sciences, New Delhi, India; 2 All India Institute of Medical Sciences, New Delhi, India; 3 All India Institute of Medical Sciences, New Delhi, India

**Keywords:** Erdheim Chester disease, multimodality imaging, *Erdheim Chester* liga, daugiamodalinis vaizdinimas

## Abstract

We hereby report a 40-year-old woman presenting with chest pain and recurrent pericardial effusion. Cardiac MRI revealed diffuse sheath-like mass involving predominantly the right heart chambers. She also had bony and perirenal involvement. Additionally, she had a pineal gland tumor, a biopsy from which revealed xanthogranulomatous infiltration with foamy histiocytes which were CD 68 positive and CD1a negative, diagnostic of Erdheim Chester disease. Erdheim Chester disease is a rare form of non-Langerhans cell histiocytosis. We highlight the importance of multimodality imaging for comprehensive assessment of all the organ systems to enable prompt diagnosis of this rare condition.

## Abstract (it)

40 metų moteris kreipėsi dėl krūtinės skausmo ir pasikartojančio perikardo efuzijos. Širdies MRT tyrimas parodė esant difuzinę apvalkalo tipo masę, apimančią daugiausia dešiniąsias širdies kameras. Taip pat buvo kaulų ir perirenalinis pažeidimas. Be to, rastas epifizės liaukos navikas, remiantis biopsija identifikuota ksantogranulomatinė infiltracija su putotais histiocitais, kurie buvo CD 68 teigiami ir CD1a neigiami, moteriai diagnozuota *Erdheim Chester* (Erdheimo Česterio) liga. Ši liga yra reta ne Langerhanso ląstelių histiocitozės forma. Reikėtų pabrėžti daugiamodalinės vaizdinės diagnostikos svarbą visoms organų sistemoms visapusiškai įvertinti, kad būtų galima greitai diagnozuoti šią retą ligą.

## Introduction

*Erdheim-Chester disease* (ECD) is a rare type of non-Langerhans cell histiocytic disorder usually characterized by the tissue infiltration by foamy histiocytes which are CD 68+ and CD1a negative. [1] This disease is frequently characterized by multi-system involvement. A systematic review by Cives et al. reported the skeletal involvement in 74%, neurologic in 25–50%, cardiovascular in 33%, dermatological in 27%, orbital in 25%, pulmonary in 18%, and retroperitoneal (including renal) involvement in 33% of the patients [2]. Patients with ECD with multi-system involvement have poor prognosis. The mean survival time after diagnosis has been reported to be 2.3 years [2]. The morbidity and mortality are worse in patients with neurological and cardiovascular involvement [3]. Given the dismal prognosis, early diagnosis and prompt management are crucial. We hereby present a case of ECD with multi-system involvement evaluated by comprehensive multi-modality imaging and histopathological examination. To the best of our knowledge, biopsy-proven accounts of ECD with such extensive multi-system involvement are rare.

## Case Report

**Figure 1:**
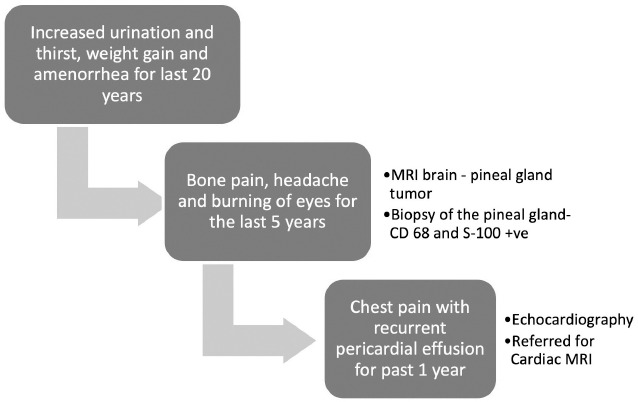
Chart depicting the timeline of symptoms and their presentation

A 40-year-old woman presented with bone pain, headache and burning of eyes for the last 5 years. MRI brain conducted earlier for these symptoms had revealed a pineal gland tumor. A subsequent biopsy of the pineal gland tumor showed xanthogranulomatous infiltration of pineal gland with histiocytes positive for CD 68 and S-100 protein but not for CD1a. On further interrogation, she also gave a history of increased urination and thirst, weight gain and amenorrhea for the past 20 years. Figure 1 highlights the sequence of the clinical presentation. At that time, the patient complained of chest pain with recurrent pericardial effusion for the past one year. Transthoracic echocardiography revealed moderate pericardial effusion with mass-like diffuse thickening along the right atrium, right ventricle wall, interatrial septum as well as aortic root. She was now referred for cardiac MRI to evaluate the diffuse thickening along the cardiac chambers.

**Figure 2:**
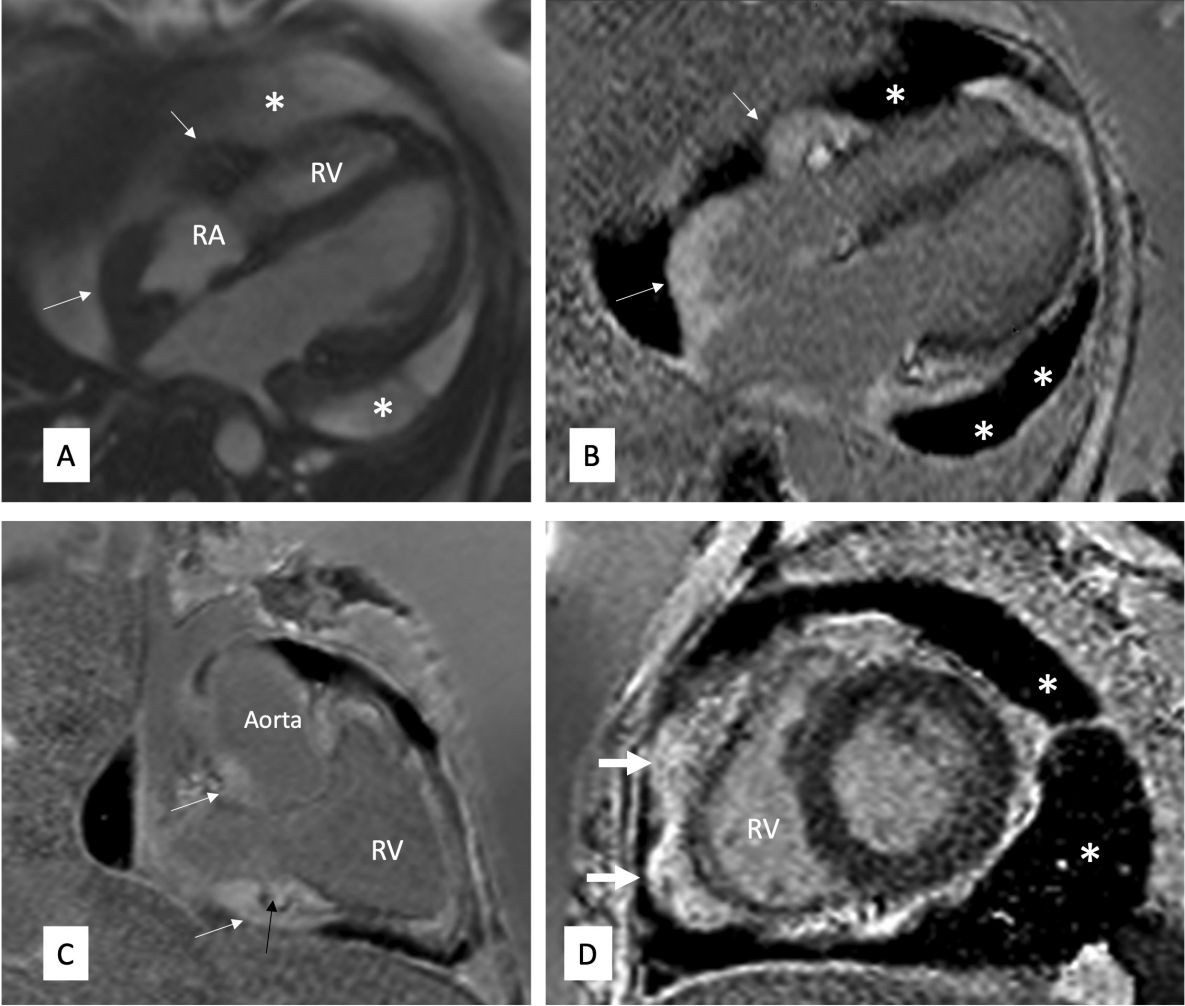
Cardiac MRI cine image in 4 chamber (A) view, late gadolinium enhancement images in 4 chamber (B), vertical long axis (C) and short axis (D) views show sheath-like thickening in the pericardium, right atrial (RA) wall, right atrioventricular groove encasing the right coronary artery (the black arrow in C), and also along the aortic root. This thickening also shows bright late gadolinium enhancement (B–D). Moderate pericardial effusion (white asterisks) is noted *Note*. RV: Right ventricle

**Figure 3:**
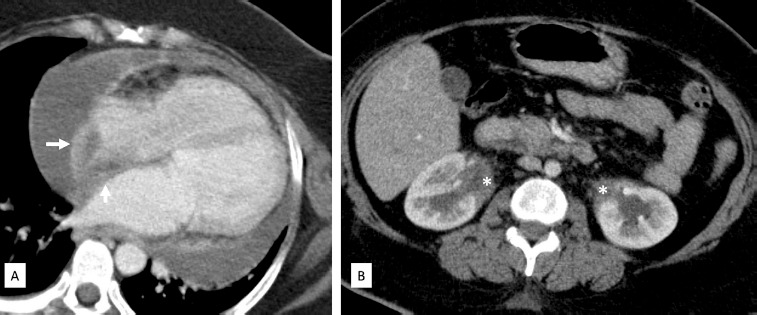
MRI brain T1 pre-contrast (A) and post-contrast (B) images in sagittal (A) and axial (B) views show a brightly enhancing lesion (the white block arrow) of a size of 2x2 cm in pineal gland which was causing aqueductal compression

**Figure 4:**
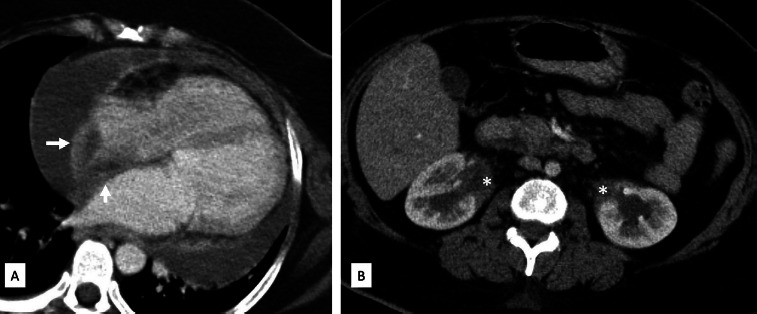
Delayed CT image in 4 chamber view (A) shows right atrial and right atrioventricular groove mass lesions which are similar to those seen on cardiac MRI. Abdominal CT axial image (B) shows bilateral perirenal soft tissue in parapelvic location (the white asterisks) encasing the renal hilar structures with resultant bilateral hydronephrosis

Cardiac MRI showed sheath-like thickening in the pericardium, right atrial wall, right atrioventricular groove encasing the right coronary artery and along the aortic root. This thickening also showed bright late gadolinium enhancement. Moderate pericardial effusion was noted (Figure 2). Concurrent MRI brain showed a T2 hypointense lesion of a size of 2x2 cm in the pineal gland which was causing aqueductal compression leading to dilatation of both the lateral and the third ventricle (Figure 3). In view of encasement of the right coronary artery (RCA) by the mass-like thickening, coronary CT angiography was also done to rule out RCA infiltration. Delayed scans were also obtained to evaluate the abdomen. CT showed RCA to be widely patent without any obstruction or infiltration of the coronary artery. Right atrial and right atrioventricular groove mass lesions were similar to those seen on cardiac MRI. Abdominal CT showed bilateral perirenal soft tissue in parapelvic location encasing the renal hilar structures with resultant bilateral hydronephrosis (Figure 4).

On further evaluation, the laboratory tests for tuberculosis, pituitary and thyroid function, as well as other immunological markers were negative. Based on CT and MRI findings as well as the biopsy findings from the pineal gland (CD68+, CD1a- histiocytes), a diagnosis of Erdheim Chester disease was made. This was further strengthened by the presence of sclerotic lesions in epiphysis of left tibia, as evidenced by hypodense lesions on T2 weighted MRI images. ^99m^Tc-MDP whole body bone scintigraphy was also implemented, which revealed an increased radiotracer uptake in the lower part of both right and left tibia. The patient was managed with diuretics and interferon treatment and was advised a regular follow-up.

## Discussion

ECD is a rare form of non-Langerhans cell histiocytosis [4]. This disease has systemic predilection and is characterized by infiltration of multiple organ systems by xanthogranulomatous infiltrates. Immunohistochemical markers are critical for definitive diagnosis. CD68 is a pan-macrophage marker found on lysosomes of tissue histiocytes. In ECD, infiltrating histiocytes strongly express CD68, reflecting their monocyte-macrophage lineage. CD1a, a glycoprotein expressed on Langerhans cells and cortical thymocytes, is the key distinguishing marker, as it is positive in LCH but negative in ECD. S-100 protein, a calcium-binding protein present in cells of neural crest origin, is typically strongly positive in LCH but negative or only weakly positive in ECD. Our patient’s biopsy showed the classic ECD pattern: CD68(+), CD1a(−), with weak S-100 positivity. This immunoprofile, combined with the characteristic foamy histiocyte morphology and xanthogranulomatous infiltration, established the definitive diagnosis of ECD and excluded LCH which is characterized by histiocytes which are positive for CD1a, but are negative for CD68, as well as other histiocytic disorders [5]. Pathological confirmation of CD68(+), CD1a(−) histiocytes is both sufficient and mandatory for the diagnosis of ECD. Imaging, histopathology and immunohistochemistry play a central role in diagnosis, treatment and follow-up [1]. Clinically, in ECD, skeletal involvement with resultant bone pain is the most common symptomatology, and the clinical triad also consists of exophthalmos and central diabetes insipidus, which is somewhat unique to ECD [6].

On imaging, close differentials of ECD include cardiac lymphoma and IgG4 related disease, both of which may be indistinguishable on imaging and may have similar organ involvement. Primary cardiac lymphoma typically presents with heterogeneous T2 signal intensity on MRI and shows mildly homogeneous to heterogeneous contrast enhancement. In contrast, our patient demonstrated bright, homogeneous *Late Gadolinium Enhancement* (LGE), which is more characteristic of ECD. Additionally, lymphoma commonly shows restricted diffusion and lacks the perirenal and skeletal involvement pattern seen in ECD. IgG4-related disease can mimic ECD both clinically and radiologically, with similar cardiac and perirenal involvement. However, IgG4-related disease shows elevated serum IgG4 levels (>135 mg/dL) and tissue IgG4/IgG ratio >40% on histopathology. Our patient had normal IgG4 levels, and the biopsy showed characteristic foamy histiocytes rather than storiform fibrosis and lymphoplasmacytic infiltration typical of IgG4 disease.

Cardiac involvement has been reported in 40–75% of patients and offers the worst prognosis. Our patient presented with chest pain with recurrent pericardial effusion. Ghotra et al. described cardiovascular imaging findings in 24 patients. In their series, pericardial involvement (13%), myocardial infiltration (25%), valvular disease (17%), aortic/vascular disease (17%), and coronary artery disease (25%) were more commonly seen [7]. A contrast-enhanced cardiac MRI is the investigation of choice to evaluate the extent of myocardial and pericardial infiltration as well as to assess ventricular functions and for tissue characterization. Differentiating myo-pericardial ECD from primary cardiac lymphoma is difficult. While lymphoma often shows a heterogenous signal and is mildly homogeneous to heterogenous contrast enhancement, the cardiac lesion in our case showed bright LGE [8].

The management of ECD is not yet fully standardized. Symptomatic care for hypertension, diabetes insipidus and bone pain is part of the comprehensive care to be undertaken. Consensus guidelines suggest that all the symptomatic patients should be put on initial therapy [9]. The management of ECD has evolved significantly with advances in molecular understanding. BRAF V600E mutations occur in approximately 50–70% of ECD patients and represent a key therapeutic target. Recent studies have demonstrated remarkable efficacy of BRAF inhibitors in this population. Specifically, *Vemurafenib*, a BRAF inhibitor, has shown objective response rates of 43–54% in BRAF-positive ECD patients, with rapid symptom improvement, often within weeks [10]. *Dabrafenib* combined with *Trametinib* (MEK inhibitor) has also shown promising results with potentially better toxicity profiles [11].

Interferon-alpha therapy remains the first-line treatment for BRAF-negative patients and those without access to targeted therapy. Response rates range from 50 to 60%, though toxicity can limit its long-term use. Recent data suggest that pegylated interferon-alpha may offer improved tolerability with similar efficacy. For patients failing conventional therapy, MEK inhibitors (*Cobimetinib, Trametinib*) have shown activity even in BRAF-wildtype disease [9].

Our patient’s multi-system involvement, and particularly the combination of cardiovascular and neurological (pineal gland) disease, places her in a high-risk category. Historical data show a median survival of 2.3 years for multi-system disease, with cardiovascular involvement being the strongest negative prognostic factor (featuring a hazard ratio of 3.5). However, contemporary series with targeted therapy report 5-year survival rates exceeding 80% in BRAF-positive patients receiving *Vemurafenib*. Given the extensive cardiac involvement with RCA encasement, this patient warrants BRAF mutation testing. If a patient is BRAF-positive, targeted therapy would be recommended over *Interferon* given the superior cardiovascular response rates. Close monitoring with serial cardiac MRI every 3–6 months is essential to assess the treatment response and guide therapy adjustments. The patient’s 20-year history of diabetes insipidus and recent development of symptomatic cardiac disease suggest disease progression, thereby making aggressive therapy initiation crucial.

Long-term management requires a multidisciplinary approach including cardiology for heart failure management, endocrinology for diabetes insipidus control, and oncology/hematology for systemic therapy coordination. Regular surveillance imaging is mandatory given the potential for CNS progression with hydrocephalus, which may require neurosurgical intervention.

## Conclusion

We hereby report a case of biopsy-proven multi-system ECD. Multimodality imaging allows for comprehensive assessment of all the organ systems to enable prompt diagnosis. Radiologists and physicians should be aware of the myriads of imaging manifestations of this condition.
